# Safety and clinical efficacy of immune checkpoint inhibitors in pediatric hepatocellular carcinoma: a case report and review of the literature

**DOI:** 10.3389/fonc.2025.1576892

**Published:** 2025-08-15

**Authors:** Xiaoting Zhong, Xuejiao Wen, Xinping Wang, Jianming Ye, Li Huang, Jing Wang, Jun Chi, Xiaoli Zeng

**Affiliations:** ^1^ Department of Oncology, The First Affiliated Hospital of Gannan Medical University, Ganzhou, Jiangxi, China; ^2^ First Clinical Medical College, Gannan Medical University, Ganzhou, Jiangxi, China; ^3^ Jiangxi “Flagship” Oncology Department of Synergy for Chinese and Western Medicine, Ganzhou, Jiangxi, China; ^4^ Jiangxi Provincial Unit for Clinical Key Oncology Specialty Development, Ganzhou, Jiangxi, China; ^5^ Jiangxi Clinical Research Center for Cancer, Ganzhou, Jiangxi, China

**Keywords:** immunotherapy, immune checkpoint inhibitors, pediatric hepatocellular carcinoma, HCC, adverse events

## Abstract

**Background:**

Pediatric hepatocellular carcinoma (HCC) is rare, with surgical resection and liver transplantation as primary treatments. No standard options exist for unresectable/metastatic disease. Although immune checkpoint inhibitors (ICIs) show efficacy in adults, their pediatric safety and efficacy remain unestablished.

**Case presentation:**

We report two cases of pathologically confirmed pediatric HCC treated with ICIs. The first patient underwent transhepatic arterial chemoembolization (TACE) and sintilimab immunotherapy. The second patient received oral sorafenib-targeted therapy followed by sequential immunotherapy with tirilizumab and sintilimab. The only adverse reaction of grade 3 or higher was skin rashes.

**Methods:**

We summarized the characteristics and treatment strategies of two pediatric HCC cases (<18 years of age) treated with ICIs at our center. We reviewed previous case reports, case series, and clinical studies on ICI treatment for pediatric HCC. All cases were evaluated for efficacy using the HCC-modified Response Evaluation Criteria in Solid Tumors every 2–3 cycles after the treatment and serial tracking of alpha-fetoprotein (AFP) levels. Treatment-related adverse reactions were graded according to the Common Terminology Criteria for Adverse Events version 5.0.

**Results:**

The first patient underwent two cycles of targeted therapy and immunotherapy, after which the tumor was assessed as having progressed. The patient then received TACE treatment and three consecutive cycles of sintilimab and lenvatinib combination therapy, resulting in stable tumor evaluation. However, after discontinuing lenvatinib, the patient’s AFP levels rose sharply, and one cycle of HAIC therapy was administered, successfully lowering the AFP levels. The second patient did not respond to immunotherapy despite the combination of targeted therapies. One patient treated with sintilimab developed a grade 3 rash, although it did not occur upon re-administration of the drug. No severe adverse reactions were observed in patients treated with tirilizumab. In the literature, most pediatric HCC cases were fibrolamellar carcinomas, which showed encouraging results after treatment with pembrolizumab, leading to longer patient survival.

**Conclusion:**

The efficacy and safety of ICIs in pediatric HCC require further validation. Ongoing prospective studies will determine their clinical role, necessitating cautious application until robust evidence emerges.

## Introduction

Hepatocellular carcinoma (HCC) is the second most common primary liver malignancy in children following hepatoblastoma (HB). Approximately two-thirds of pediatric HCC cases occur in individuals aged 15–19 years, accounting for 87% of liver tumors in this age group (1). Pediatric HCC is classified into two main types based on histology: conventional hepatocellular carcinoma (cHCC) and fibrolamellar carcinoma (FLC). FLC is rare and aggressive, with an incidence of approximately 1 in 5 million, accounting for 1–9% of all HCC cases. FLC presents differently from cHCC and predominantly affects adolescents and young adults (2). In Asian children, HCC often develops in the setting of chronic or congenital liver disease.

While collaborative frameworks exist for pediatric liver tumors, standardized therapeutic algorithms specifically for HCC remain limited. These protocols predominantly address HB, with systemic therapy recommendations for pediatric HCC lacking robust evidence-based support, particularly regarding functional systemic agents such as immune checkpoint inhibitors (ICIs). Due to the low incidence of pediatric HCC, there are no standardized treatment guidelines. Retrospective studies suggested that cHCC might be sensitive to chemotherapy, with surgical resection and liver transplantation being the main curative treatments (3, 4). For resectable HB involving no more than three segments of the liver, surgical treatment combined with neoadjuvant (or adjuvant) chemotherapy can achieve a long-term disease-free survival rate of approximately 85-90% (5). The emergence of immune checkpoint inhibitors (ICIs) dramatically changed the landscape of cancer treatment. However, pediatric patients with HCC have been frequently excluded from ICI clinical trials, leading to a lack of data on the safety and efficacy of immunotherapy in this population. Therefore, ICI treatment for pediatric HCC has only been reported in a few cases and case series. This study presents two cases of pediatric HCC diagnosed at our center and includes a systematic search and review of the existing literature to explore the potential of ICIs for treating pediatric HCC.

We collected data on two pediatric HCC cases diagnosed at the First Affiliated Hospital of Gannan Medical University between January 2020 and December 2023. Both patients received immunotherapy. This study aimed to detail the characteristics, treatment modalities, and outcomes of these cases; assess the adverse effects of ICIs; and evaluate their efficacy when combined with local therapy.

## Methods

### Clinical data

In this study, we retrospectively analyzed the clinical characteristics of pediatric HCC and assessed the efficacy and adverse effects of treatment with ICIs. Currently, approximately 12 cases utilizing ICIs for treating pediatric HCC have been reported. We collected data on age, pathological type, ICI type, dosage, treatment duration, combined treatment regimen, adverse reactions, and therapeutic effects. Additionally, we included clinical characteristics from two pediatric patients with HCC post-ICI treatment in our hospital. Since the ALBI score was able to more objectively assess the degree of hepatic impairment in non-cirrhotic patients compared to the Child-Pugh score, and considering the lack of relevant guidelines or consensus on pediatric HCC at present, we used the ALBI score to reflect patients’ liver function. This study was approved by the Ethics Committee of the First Affiliated Hospital of Gannan Medical University. Clinical data were collected by reviewing the case information, including clinical symptoms, imaging findings, and follow-up results.

### Case presentation

#### Case 1

**Figure 1:**
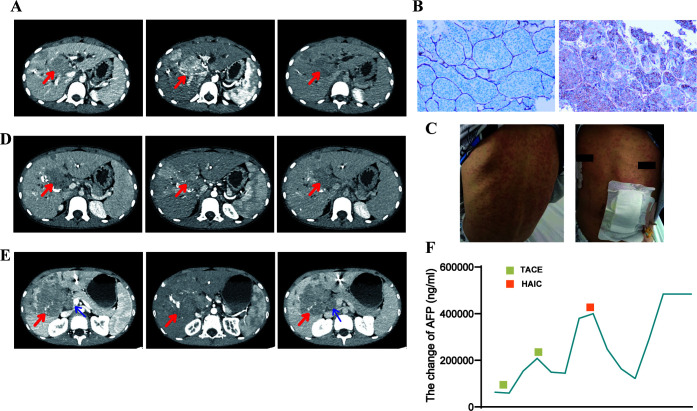
Pre- and post-treatment CT images, pathology images, adverse reactions, and alpha-fetoprotein (AFP) changes in patient 1. **(A)** Enhanced computed tomography (CT) scan before treatment showing multiple lesions in the liver parenchyma with significant fast-in and fast-out signs. **(B)** Conventional hepatocellular carcinoma (cHCC) is the most likely diagnosis based on histology, hematoxylin-eosin (HE) staining morphology, and immunohistochemistry (HE x40, CD34+ on the left, AFP+ on the right). **(C)** The patient developed large, red rashes on his torso following sintilimab treatment. **(D)** Follow-up CT scan, taken 1 month after TACE treatment, revealing iodized oil deposits within the lesion and a reduction in tumor volume compared to pre-treatment. **(E)** Follow-up CT showing significant tumor enlargement and additional invasion of the pancreatic head and portal vein trunk after two cycles of sintilimab and bevacizumab regimen, indicating tumor progression. **(F)** AFP levels significantly decreased after receiving TACE and HAIC therapies, as shown in the trend of AFP changes.

The first case involved an 11-year-old boy who presented with generalized jaundice, itching, and a history of seropositive hepatitis B virus (HBV) infection with an unknown viral load. Both his mother and grandmother had a history of HBV. Initial laboratory tests on admission are shown in **Table 1**. Enhanced abdominal computed tomography (CT) scan suggested HCC with multiple intrahepatic metastases; magnetic resonance imaging (MRI) confirmed the diagnosis while also detecting thrombosis in the right branch of the portal vein. CT images (**Figure 1**) showed a mass of approximately 3.4 cm × 5.6 cm in the portal area of the liver, with typical HCC characteristics of “fast in and fast out.” Percutaneous transhepatic cholangiogram drainage (PTCD) was performed to relieve the patient’s obstructive jaundice. Subsequently, histopathological diagnosis of HCC was confirmed by percutaneous liver biopsy (**Figure 1B**). The immunohistochemical results were as follows: Arginase-1 (weak +), AFP (+), Gypican-3 (+), CK7 (-), CK20 (-), CK19 (-), HSP7 0 (partially +), Ki-67 (hot spot area about 50% +), CD10 (-), CD34 (vascular +), Vim (-), CK (+). Combined with the hematoxylin-eosin staining morphology and immunohistochemical findings, the pathological type was considered to be cHCC. The patient also had elevated serum alpha-fetoprotein (AFP), and glycan antigen-199 levels. The patient’s liver function was classified as grade 2 using the Albumin-Bilirubin (ALBI) score, and the disease was staged as Barcelona Clinic Liver Cancer Stage C. After evaluation, it was concluded that the patient failed to meet the indications for surgical treatment as well as for liver transplantation. Total bilirubin was significantly decreased after PTCD. Following this, the patient underwent transhepatic arterial chemoembolization (TACE) and was subsequently treated with sintilimab (100 mg d1) combined with bevacizumab (400 mg d1). After 1 week of treatment, the patient developed a high fever and a generalized rash affecting the face, neck, trunk, bilateral thighs, and buttocks (**Figure 1C**), which was diagnosed as an adverse reaction to sintilimab (Grade 3). The occurrence of moderate to severe immune-related adverse events may impair organ function, leading to a reduced quality of life and even life-threatening conditions. The American Society of Clinical Oncology guidelines indicate that when patients experience an immune-related adverse reaction of grade 3 or higher, the medication needs to be suspended for treatment. After the adverse events were reduced to grade 1 or below, immunotherapy could be attempted again depending on the patient’s condition and expected efficacy. Therefore, we referred to the guidelines for the management of treatment-related toxicity of ICIs, which have been updated based on the results of the most recent studies, to treat this patient who developed rashes on the line (6). The initial anti-allergic treatment with loratadine was ineffective, and considering the patient’s high persistent fever, methylprednisolone (0.5–1 mg/kg/day) was administered. This treatment successfully normalized the patient’s temperature and led to the gradual resolution of the rash. Follow-up CT scans suggested that the liver tumor had reduced in size (about 3.2 cm × 4.6 cm) and that the enhancement had diminished (**Figure 1D**). We considered the current treatment program to be effective. As the adverse effects of the rash subsided and the patient had no fever, we reinstated the patient’s original regimen after a thorough evaluation of the risks and benefits of the treatment. Subsequently, the patient continued with one cycle of sintilimab and bevacizumab regimen without local treatment. However, after two cycles of this regimen, follow-up CT showed the tumor to be approximately 6.8 cm × 6.6 cm in size, and there was additional invasion of the pancreatic head and portal vein trunk (**Figure 1E**), which indicated tumor progression. Additionally, the patient’s AFP level was significantly elevated. Therefore, the patient underwent another session of TACE and was then treated with three consecutive cycles of sintilimab in combination with lenvatinib. The ICI (sintilimab) was not changed after progressive disease (PD) was determined, primarily due to the patient’s family’s financial constraints and their enrollment in a sintilimab patient assistance program (which provided the drug free of charge). This treatment led to a slight tumor reduction on follow-up and an overall assessment of stable disease (SD). The patient later discontinued lenvatinib due to recurrent vomiting. Within 1 month, AFP levels sharply increased, prompting a switch to hepatic arterial infusion chemotherapy (HAIC) by continuous arterial infusion using the mFOLFOX regimen (oxaliplatin 85 mg/m^2–^2 h + leucovorin 400mg/m^2–^2 h + 5-fluorouracil 400 mg/m^2^ intravenously 10 min + 5-fluorouracil 1200 mg/m^2^ 23h). Other than fatigue, vomiting, or nausea, no adverse reactions were observed. A significant decrease in AFP levels was observed 1 month after starting HAIC (**Figure 1F**), and immunotherapy with sintilimab was continued for two more cycles. However, after discussions regarding the risks, benefits, and prognosis, the patient’s parents subsequently declined further surgical intervention and antitumor therapy on behalf of the child. Two months later, the patient presented with significant tumor progression and liver failure, ultimately leading to death from gastrointestinal bleeding 9 months after the initial diagnosis.

**Table 1:** Patient’s laboratory test and examinations results.

Laboratory tests (units)	Patient 1	Patient 2	Reference range
White blood cell (*10^9^/L)	8.53	5.55	3.5-9.5
Hemoglobin (g/L)	132	107	130-175
Platelet (*10^9^/L)	245	477	125-350
PT (seconds)	11.3	13.1	9-13
Serum albuming(g/L)	42.3	42.3	40-55
Alpha fetoprotein(ng/L)	62971	>484000	<7
HBV-DNA (IU/ml)	2.00×10^5^	1.14×10^3^	negative
Total bilirubin(µmol/L)	290.9	21.4	<26
Direct bilirubin(µmol/L)	253.8	11.1	<8
ALT (U/L)	164	131	9-50
AST (U/L)	157	405	15-40
ascites	Slight	Absent	–
encephalopathy(grade)	None	None	–

PT, Prothrombin time; AFP, Alpha fetoprotein; HBV, Hepatitis B virus; AST, Aspartate aminotransferase; ALT, Alanine aminotransferase.

#### Case 2

**Figure 2:**
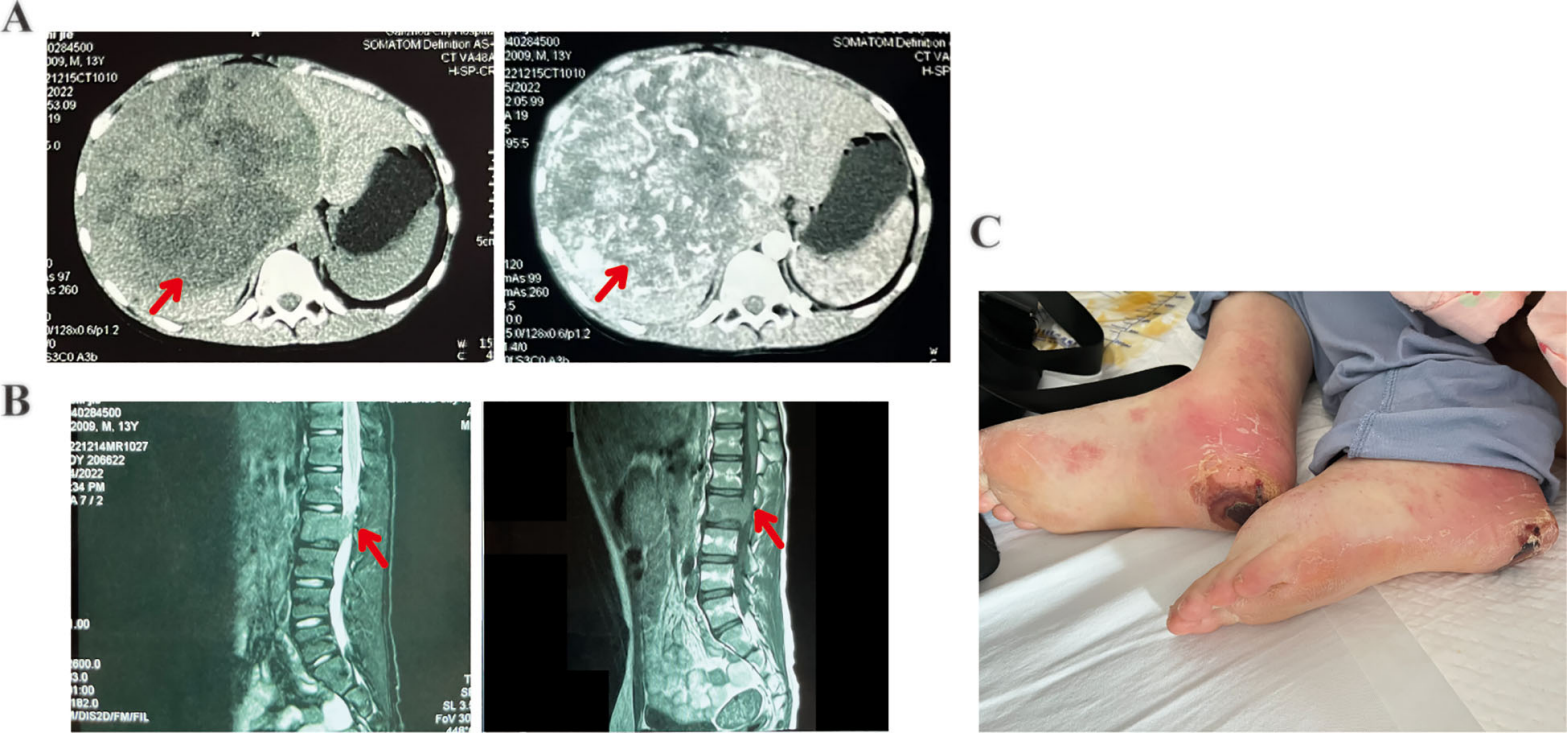
Patient 2 computed tomography (CT) images, magnetic resonance imaging (MRI) findings, and post-treatment adverse events. **(A)** Abdominal contrast-enhanced CT image showing multiple nodules in the liver and a primary hepatocellular carcinoma (giant type) in the right hepatic lobe. **(B)** Lumbar MRI showing osteolytic bone degradation of the L2 vertebra and a localized soft tissue mass extending into the spinal canal and in contact with the spinal cord (left: T2-weighted image; right: T1-weighted image). **(C)** Desquamation, ulceration on the heels, and scattered erythema and nodules on both lower extremities (hand-foot skin reaction).

The second case involved a 14-year-old boy with a history of being an HBV carrier (HBsAg+, HBeAg+, and HBcAb +), whose mother was also an HBV carrier. The patient was admitted to a local hospital with progressive pain and numbness in both lower limbs for over 2 weeks and underwent a contrast-enhanced abdominal CT examination. The scan revealed a giant primary HCC in the right lobe of the liver, multiple nodules (**Figure 2A**), and L2 vertebral metastasis. Additionally, an abnormal signal in the L2 vertebra was noted on the lumbar and sacroiliac joint MRI, suggesting the possibility of metastatic lesions. The lesion had invaded the spinal canal and was not visible in the spinal cord (**Figure 2B**). The patient was diagnosed with moderately differentiated HCC (FLC or cHCC not differentiated) based on a liver aspiration biopsy at another hospital. Upon arrival at our hospital, the patient complained of back pain and difficulty walking, requiring him to sit at all times. Physical examination revealed pressure sores on the sacrum, grade 1 muscle strength in both lower extremities, mild pitting edema in both lower extremities, pressure ulcers on the soles, and redness and exudative signs in both ankles. Due to spinal cord compression, the patient was unable to care for himself, and his performance status score was 3. Laboratory findings (**Table 1**) indicated a grade 1 ALBI score and Barcelona Clinic Liver Cancer stage C. Following diagnosis, the patient started sorafenib (1 tablet twice daily). After 2 weeks, the patient developed skin itching, scattered pale erythematous spots on the neck, chest, and limbs, flaking, and ulceration of the heels (**Figure 2C**). These symptoms were considered to be associated with sorafenib treatment and cleared up after discontinuing the drug. Due to pain in both lower limbs from spinal cord compression, radiation therapy was added to alleviate the symptoms, with a total dose of 3000 cGy, which significantly relieved the pain. After discontinuing sorafenib-targeted therapy due to adverse events, the patient underwent two cycles of immunotherapy with tislelizumab (100 mg d1). This was later switched to three cycles of sintilimab (100 mg) plus bevacizumab (300 mg) following assessment of disease progression at another hospital. The efficacy evaluation showed disease progression without a reduction in AFP levels. AFP was consistently greater than the upper limit of detection throughout treatment and changes could not be assessed. Later, the patient developed multiple bone metastases and a pathological fracture. The patient passed away 11 months after the initial diagnosis. The adverse reactions included rashes (grade 2), hand-foot skin reactions, nausea, vomiting (grade 1), and leukopenia (grade 1).

### Literature search

The PubMed and the CNKI database were searched from its inception to July 2024, using the keywords “Pediatric,” “children” and “Hepatocellular Carcinoma” (excluding “Hepatoblastoma”). Literature on the treatment of pediatric HCC was reviewed. We included pediatric patients with clinically or pathologically confirmed HCC and excluded cases where ICIs were not used in treatment. The authors carefully reviewed each patient’s information and excluded duplicates. Data on patient age, sex, type of pathology, type of ICIs drugs, dosage, treatment duration, combination therapy regimen, adverse effects, and treatment outcomes were collected. Details of the 12 reported pediatric HCC cases are shown in **Table 2**.

**Table 2:** Case reports and case series of children, adolescents with hepatocellular carcinoma (HCC) treated with the currently available ICIs.

Author (Reference)	ICI	Cycles (ICI)	Combination treatment	Histopathologic	Gender	Age (Years)	Dose	Adverse events	Response (ICI)	OS
Dasgupt A, et al. 2022 (25)	nivolumab	6	surgical resection, pegylated interferon alpha, capecitabine	FLC	female	14	NR	T1DM, DKA	SD	NA
Kang E, et al., 2022 (24)	Pembrolizumab	3	three cycles of PLADO,DEB-TACE with doxorubicin,surgical resection,Transplantation	NFL-HCC	male	14	2mg/kg every 3 weeks	no side effects	PD	NA
Ugonabo O, et al., 2023 (14)	atezolizumab	NR	bevacizumab	FLC	male	adolescents	NR	NR	NR	NA
O’Neill AF, et al., 2024 (15)	pembrolizumab	25	Radiotherapy, liver transplantation	FLC	male	17	2mg/kg every 3 weeks	immunogenic hepatitis (Grade 3),immune-mediated pneumonitis (Grade 2)	CR	NA
	Pembrolizumab + nivolumab/ipilimumab	15+2	RFA,synthetic peptide vaccine, Radiotherapy, bevacizumab	cHCC	male	14	2mg/kg every 3 weeks	hypothyroidism (Grade 2)	SD	8 Years
	Pembrolizumab, nivolumab/ ipilimumab	49	Radiotherapy	FLC	female	15	2mg/kg every 3 weeks	loose stools (Grade 1), intermittently elevated lipase (Grade 1)	SD	6 Years
	pembrolizumab	10	Yttrium-90	FLC	male	16	2mg/kg every 3 weeks	no side effects	PD	2Years
	pembrolizumab	7	–	FLC	male	12	2mg/kg every 3 weeks	loose stools (Grade 2), elevation of liver enzymes (Grade 1)	PD	2Years
	Pembrolizumab + nivolumab/ipilimumab	2+1	–	FLC	male	16	2mg/kg every 3 weeks	no side effects	PD	3Years
Geoerger B, et al., 2020 (35)	pembrolizumab	NR	NR	HCC	NR	NR	2mg/kg every 3 weeks	NR	PD	NR
	pembrolizumab	NR	NR	HCC	NR	NR	2mg/kg every 3 weeks	NR	PD	NR
	pembrolizumab	NR	NR	HCC	NR	NR	2mg/kg every 3 weeks	NR	PD	NR

ICI, Immune Checkpoint Inhibitor; FLC, fibrolamellar hepatocellular carcinoma; T1DM, type1 diabetes mellitus; DKA, diabetic ketoacidosis; NR, not reported; AE, adverse events; SD, stable disease; PR, partial response; CR, complete response; PD, progressive disease; NA, not achieved; NOS, not otherwise specified; cHCC, conventional hepatocellular carcinoma; NFL-HCC, non-fibrolamellar hepatocellular carcinoma; PLADO, cisplatin/doxorubicin/dexrazoxane; DEB-TACE, drug-eluting bead transarterial chemoembolization; RFA, radiofrequency ablation.

### Follow-up

We collected data on adverse reactions and the efficacy of ICIs in pediatric patients with HCC by reviewing clinical case data. All cases were evaluated as having SD, partial response (PR), complete response (CR), or PD using the Modified Response Evaluation Criteria in Solid Tumors. Treatment-related adverse reactions were graded according to the Common Terminology Criteria for Adverse Events version 5.0. The clinical histories of patients were obtained through a retrospective chart review.

## Results

Seven of the 12 pediatric patients with HCC were histopathologically classified as having FLC, one as cHCC, while the pathological type was not specified for three others. Among the seven pediatric patients with HCC treated only with pembrolizumab, six underwent efficacy evaluations of PD. Of these, one patient with FLC achieved a CR after combining radiotherapy and liver transplantation. Pembrolizumab combined with nivolumab/ipilimumab prolonged survival in some cases, with three patients having an overall survival of 8, 6, and 3 years, two of whom achieved SD. One male pediatric patient with HCC treated with nivolumab in combination with surgical resection, pegylated interferon-alpha, and capecitabine also showed SD. One patient treated with atezolizumab had unknown efficacy. The adverse reactions observed after ICI treatment included loose stools (2/12), grade 2 hypothyroidism (1/12), grade 1 intermittent lipase elevation (1/12), grade 1 elevated liver enzymes (1/12), type 1 diabetes mellitus (1/12), and diabetic ketoacidosis (1/12). Remarkably, one patient developed immune hepatitis (grade 3) and pneumonitis (grade 2) after treatment.

## Discussion

Pediatric HCC is the second most common malignant liver tumor in children, following HB. Perinatally acquired HBV infection, hepatorenal tyrosinemia, progressive familial intrahepatic cholestasis, glycogen storage disease, Alagille syndrome, and congenital portosystemic shunt are important predisposing factors (7). In Asia, most pediatric HCC cases are linked to HBV infection, often from mother-to-child transmission. Therefore, vaccination is crucial for disease prevention and should be emphasized. It is commonly known that both the HBV infection and the HCC itself lead to impaired liver function, and the degree of liver function not only affects the efficacy of cancer treatment but is also an independent prognostic factor in patients with HCC (8). Although the Child-Pugh score, which includes albumin levels and prothrombin time, serum bilirubin, ascites, and encephalopathy, is widely used in various staging systems for HCC, the accuracy of the Child-Pugh score in the assessment of noncirrhotic disease is doubtful as it may not exactly reflect the hepatic function in this group of patients. In recent years, the ALBI grade has been proposed as a new assessment method considered to be able to objectively and reproducibly measure the liver functional reserve in patients with HCC (9, 10). The ALBI grade defines 3 levels (I to III) of liver impairment using the albumin and bilirubin levels, both of which can be easily obtained from patient blood samples. The ALBI grade had been found to be associated with patient survival, tumor recurrence, and liver failure after hepatectomy (11). In pediatric patients with HCC, there is no standard protocol for the evaluation of liver function. The ALBI score was selected for liver function assessment based on its superior objectivity in non-cirrhotic settings. Therefore, we ultimately chose the ALBI score as a measure of liver function in in this study.

Pediatric HCC is classified into two histological types: cHCC and FLC. While cHCC typically involves mutations in TP53 and β-catenin (CTNNB1), FLC is characterized by the activation of protein kinase A, most commonly via a DNAJB1-PRKACA fusion transcript, secondary to a somatic intrachromosomal deletion on chromosome 19 (12). Pediatric cHCC may be more sensitive to chemotherapy than that in adults, and chemotherapy has been the main treatment modality for advanced cases in children. Due to limited research, treatment regimens for advanced pediatric HCC are often adapted from adult guidelines. In the phase III HIMALAYA study (NCT03298451) of unresectable HCC, investigators randomly assigned adult patients with inoperable HCC who met inclusion criteria to the Single Tremelimumab Regular Interval Durvalumab (STRIDE) group (n = 393), the durvalumab group (n = 389), or the sorafenib group (n = 389). After long-term follow-up, the study found that STRIDE significantly improved overall survival compared to sorafenib alone. The study concluded that this combination regimen strongly stimulated T-cell cloning, which released T-cells from their immunosuppressive state and stimulated the patient’s own immune system to attack the tumor cells (13). In addition, previous studies demonstrated that combining PD-1 inhibitors with bevacizumab was an important treatment option in inoperable HCC without prior systemic therapy (14, 15). PD-1 inhibitors stimulate T-cell activity by blocking the interaction between two proteins, thereby promoting the activation of toxic T-lymphocytes to enhance the body’s immune response against tumors (16). The IMBrave 150 study found that atezolizumab in combination with bevacizumab significantly improved survival in adult patients with HCC compared to sorafenib (15). In a randomized phase II-III clinical study (ORIENT-32) conducted in China, investigators confirmed that sintilimab in combination with bevacizumab showed significantly greater overall and progression-free survival compared to sorafenib in the treatment of adult patients with HCC (14). This combination regimen could provide a new treatment option for patients with HCC. However, there were certain differences between the treatment of children and adults with ICIs. The efficacy and safety of ICIs in pediatric HCC patients need to be evaluated comprehensively in conjunction with their unique physiological and pathological characteristics (17). There were significant differences between children and adults in terms of liver metabolic capacity and immune system development. Studies have shown that children may have a higher clearance rate of ICIs, but the manifestations of immune-related adverse events are more diverse (18). This difference may be related to the high reactivity of the pediatric immune system, and dose adjustments may be considered (18). Therefore, we treated our two cases using appropriate dosage adjustments and closely monitored for adverse effects by referring to current adult guidelines and clinical studies. On the basis of efficacy, safety, and economic considerations, the less expensive and proven potentially effective regimen, sintilimab, in combination with bevacizumab, was chosen for both patients. Given that sintilimab had been procured by the families through a patient assistance program (with non-refundable provisions), and considering the absence of severe immune-related adverse events or hyperprogression, the multidisciplinary tumor board recommended maintaining sintilimab while intensifying local therapy for disease control.

There are no established therapeutic recommendations for FLC, even for adults. Recent cases suggested that although the overall survival of patients with cHCC who had completed follow-up was longer than that of patients with FLC, FLC might respond more favorably to immunotherapy compared to cHCC (19). For instance, a pediatric patient with FLC treated with pembrolizumab achieved SD after postoperative recurrence. Upon developing lung metastases, the patient was retreated with pembrolizumab combined with radiation therapy, ultimately achieving a CR which lasted for 18 months. The patient underwent liver transplantation for hepatic insufficiency and remained in remission 2 years post-transplantation (20). The feasibility of ICIs as a bridge therapy for liver transplantation is a current research hotspot. Recent studies have shown that PD-1 inhibitors can reduce the tumor stage of 30-40% of adults with advanced HCC, making them eligible for liver transplantation (21). However, the incidence of post-transplant rejection is as high as 39%, which may be related to high PD-L1 expression in the graft (22). Unfortunately, data on children are extremely limited. As of the reporting period, the patient was still alive. This suggested that ICIs may be an option for pre-transplant downregulation therapy in pediatric HCC patients. Overall survival for this patient had not been updated. Notably, this patient was reintroduced to immunotherapy after presenting with grade 3 immune hepatitis. However, he subsequently developed grade 2 immune pneumonitis, leading to discontinuation of treatment. This indicated that the combination of immunotherapy with other treatment modalities appeared to result in a greater clinical benefit. Particularly for patients suffering from HCC with portal vein thrombosis, who have poor prognosis, with a median survival of only 3 months when untreated (16), studies showed that combination therapies are especially important (23, 24). A propensity score-matched study found that the combination of targeted therapy and immunotherapy with TACE-HAIC significantly improved the clinical benefit for patients with HCC and portal vein tumor thrombosis versus TACE alone (25). Therefore, we implemented for pediatric HCC patients who were not candidates for curative resection or liver transplantation at our center. From our first patient, we observed that immunotherapy alone resulted in only SD. However, the patient’s AFP levels dropped significantly after adding TACE and HAIC, with the most notable decrease occurring after HAIC. Ultimately, this patient had an overall survival of 9 months. Several studies have shown that AFP is an independent prognostic biomarker and is involved in biological pathways that inhibit apoptosis and promote proliferation, migration, invasion, and metastasis of HCC cells (26, 27). AFP has been proved to block phagocytosis by triggering the polarization of M0 macrophages to an M2-like phenotype via the PI3K/Akt pathway (28). In addition, AFP hindered the proliferation of NK cells and T lymphocytes, contributing to the immune escape of HCC cells (29). Consequently, we considered that this patient’s treatment achieved a certain efficacy in combination with HAIC. This suggests that chemotherapy might be more effective than immunotherapy for pediatric HCC, in contrast to what has been typically observed in adults. A previous report discussed the use of TACE and ablation therapy in pediatric HCC (30) without referencing HAIC as a regimen. Given the sensitivity of pediatric HCC to chemotherapy, combining HAIC with immunotherapy could be a promising therapeutic strategy, although it requires full patient compliance. In terms of reported cases (19, 20, 31, 32), the efficacy of ICIs in pediatric patients with HCC appears promising and warrants further investigation in prospective clinical trials.

Based on the literature, pediatric patients with HCC typically demonstrate a median overall survival (OS) exceeding 2 years, although most cases reviewed here showed an efficacy rating of PD. Downregulation of gene expression associated with T-cell dysfunction, inflammation, cancer growth, and invasion might significantly influence immunotherapy response and disease control (20). The outcomes of our two patients were significantly worse compared to the reported literature despite receiving ICIs. This discrepancy might be related to the pathological type of the tumors and the multidisciplinary approach, as FLC was more common in literature reports, and some patients received surgical resection, liver transplantation, radiotherapy, and ablation. The variance could also stem from problems with treatment adherence since pediatric patients often struggle with consistent medication use, resulting in fewer cycles of immunotherapy. Another factor might be the difference in ICIs drugs used. Although we administered an anti-PD-1 similar to pembrolizumab, structural variations among these drugs could affect disease control rates, as a study showed that pembrolizumab has superior objective response and disease control rates compared to nivolumab (33). Unfortunately, there was limited data available for a comprehensive multi-factor analysis to address this question. However, it has been indicated that pediatric cancer patients with mismatch repair deficiencies and *SMARC1B* deficiency, which resulted in higher neoantigen burdens or increased immune cell infiltration, might show superior efficacy with ICIs (34). Whether the differing efficacy observed between these two patients and those reported previously was related to mutational load remains to be explored, as our patients were not extensively examined for these factors.

When considering immunotherapy in children, it is crucial to address potential adverse effects alongside therapeutic efficacy. While ICIs enhance immune responses and increase T-cell activity against tumor cells, they may cause immune-related adverse events such as dermatitis, pneumonia, fatigue, anemia, and thyroid dysfunction. Although immunotherapy is generally associated with lower long-term toxicity compared to chemotherapy and radiation therapy, short-term adverse events like fever, headache, rash, and chills were common, with severe complications including toxic epidermal necrolysis, autoimmune toxicity, irreversible neurotoxicity, and endocrine toxicity. The incidence and severity of these effects often depend on the drug dose and individual factors (35–37). Research on PD-1/PD-L1-specific ICIs in pediatric patients is sparse (36, 38–40). The current data did not definitively indicate whether the types, grading, and management of adverse effects in adults can be applied to children. We therefore provisionally graded immunotherapy-related adverse reactions occurring in pediatric patients with HCC according to the Adverse Event Severity Grading Criteria version 5.0. No child-specific, life-threatening adverse effects had been observed to date. Furthermore, using immunotherapy as a bridge to liver transplantation has not been linked to an increase in surgical or postoperative complications. However, reinitiating immunotherapy after a grade 3 or higher hepatic or pulmonary immune response should be performed with caution. Pembrolizumab was generally administered at 2 mg/kg every 3 weeks. In a phase I clinical study (41), sintilimab was well-tolerated in children, with no dose-limiting toxicities reported, even at the highest dose of 10 mg/kg. The frequency and nature of adverse events associated with the drug in this study were consistent with those documented in adults, and weight-based dosing proved safer than uniform dosing for individuals. Thus, we referred to this administration dose in our two pediatric patients with HCC treated with sintilimab.

The use of ICIs in pediatric tumors presents challenges for the conduction of large-scale clinical studies to determine their efficacy and safety, as is done for adults. Most existing research on immunotherapy in children has focused on hematological malignancies (42). However, there are ongoing studies investigating nivolumab and atezolizumab for solid tumors like sarcoma, neuroblastoma, and melanoma. Studies involving nivolumab, ipilimumab, atezolizumab, bevacizumab, and pembrolizumab for pediatric HCC are underway, but no new data have been published yet (ClinicalTrials.gov ID NCT05302921, NCT05468359, and NCT04134559). The ICIs used in the two cases reported here had not been clinically examined for pediatric HCC. There is a phase I clinical study of sintilimab in pediatric solid tumors, and a comparable study of anti-PD-1 camrelizumab in HB is currently being conducted (ClinicalTrials.gov ID: NCT05322187). While immunotherapy holds promise, further clinical studies are essential to provide sufficient evidence for the application of ICIs in treating pediatric HCC.

## Conclusion

Pediatric HCC generally has a poor prognosis, and no standard treatment options are currently available. ICIs are effective in treating pediatric HCC and prolonged survival to a certain extent. The combination of topical therapy with ICIs may improve clinical benefit in pediatric patients with HCC. The application of ICIs is relatively safe, and the adverse effects that occur can be treated. While immunotherapy shows promise as a treatment modality, its applicability to pediatric HCC still requires further clinical data. Collecting more cases will be crucial to providing stronger evidence for the use of immunotherapy in pediatric patients with HCC.

## Data Availability

The original contributions presented in the study are included in the article/supplementary material. Further inquiries can be directed to the corresponding author.
